# Association between micronutrients and maternal leukocyte telomere length in early pregnancy in Rwanda

**DOI:** 10.1186/s12884-020-03330-y

**Published:** 2020-11-13

**Authors:** Etienne Nsereko, Aline Uwase, Claude Mambo Muvunyi, Stephen Rulisa, David Ntirushwa, Patricia Moreland, Elizabeth J. Corwin, Nicole Santos, Jue Lin, Jyu-Lin Chen, Manasse Nzayirambaho, Janet M. Wojcicki

**Affiliations:** 1 College of Medicine and Health Sciences School of Health Sciences, University of Rwanda, P.O. Box: 3538, Kigali, Rwanda; 2 College of Medicine and Health Sciences School of Medicine and Pharmacy, University of Rwanda, P.O. Box: 3538, Kigali, Rwanda; 3 Emory University, Nell Hodgson Woodruff School of Nursing, Atlanta, Georgia USA; 4 Columbia University School of Nursing, New York, NY 10032 USA; 5 University of California San Francisco, Institute for Global Health Sciences, San Francisco, USA; 6 Department of Biochemistry and Biophysics, University of California San Francisco, San Francisco, USA; 7 Departmentof Family Health Care Nursing, University of California San Francisco, San Francisco, USA; 8 University of Rwanda College of Medicine and Health Sciences School of Public Health, P.O. Box: 3538, Kigali, Rwanda; 9 Department of Pediatrics, University of California San Francisco, San Francisco, USA; 10 Department of Epidemiology and Biostatistics, University of California, 550 16th Street, San Francisco, CA 941558 USA

**Keywords:** Nutrition, Infection, Pregnancy, Oxidative stress, Leukocyte Telomere length

## Abstract

**Background:**

Exposure to environmental stressors can lead to shorter leukocyte telomere length and increase the risk of chronic diseases. Preservation of leukocyte telomere length by reducing oxidative stress exposure and reinforcing immunity may be a mechanism by which nutritional factors delay or prevent chronic disease development.

**Methods:**

Healthy pregnant women (aged 18–45 years) at 9–15 weeks of gestation living in Gasabo District, Kigali, Rwanda, were recruited from 10 health centers for a prospective, longitudinal study from September to October 2017 to determine possible associations between nutrition health, infectious disease and leukocyte telomere length. Anthropometric and laboratory measurements were performed using standard procedures; sociodemographic parameters and health histories were assessed via surveys, and leukocyte telomere length was assessed using quantitative PCR expressed as the ratio of a telomeric product to a single-copy gene product (T/S).

**Results:**

Mean gestational age of participants (*n* = 297) at enrollment was 13.04 ± 3.50 weeks, age was 28.16 ± 6.10 years and leukocyte telomere length was 1.16 ± 0.22 (T/S). Younger age; no schooling vs. primary schooling; and lower levels of ferritin, soluble transferrin receptors and retinol-binding protein were independent predictors of longer telomere length in multivariable models.

**Conclusions:**

Leukocyte telomere length is an indicator of biological aging in pregnant Rwandan women. Maternal micronutrient status, specifically lower ferritin, soluble transferrin receptor levels, and retinol-binding protein levels were associated with longer maternal telomere length in contrast with some studies from North America and Europe. There were no associations between inflammation and infectious disease status and maternal leukocyte telomere length. Further studies are needed to enhance our understanding of the interplay between maternal nutritional status and infectious disease in relation to leukocyte telomere length in developing countries.

## Background

### Leukocyte telomeres and maternal health

Telomeres are protective nucleoprotein structures that appear at the ends of chromosomes and confer protection against chromosomal damage [1]. They undergo attrition with each cell division due to limitations in the ability of the DNA replication machinery to replicate chromosome ends. Changes in telomeres serve as a biological marker for cellular aging, [2] with critical shortening resulting in cellular senescence [3]. Telomeres shorten throughout the normal aging process, and shorter telomere length has been associated with multiple chronic diseases, including cancer, type II diabetes, and cardiovascular disease [4]. Further, they are particularly sensitive to reactive oxygen species (ROS) damage; exposure to chronic or acute and social and environmental stressors can often lead to increased ROS exposure [3, 5].

Antioxidants, which maintain homeostasis via affecting redox status and/or redox-sensitive signaling pathways and gene expression, may help reduce ROS damage [5]. They play a specific role in boosting immune response and restricting pathological aspects of the cytokine-mediated response [6, 7]. Shorter leukocyte telomere length is reportedly associated with micronutrient deficiencies and inadequate diet attributed to the insufficient intake of iron, vitamin B12 and D, and other vitamins and minerals [8, 9]. During pregnancy, maternal nutritional health is pivotal as deficiencies of certain vitamins or micronutrients and poor dietary intake may affect fetal telomere length, consequently having an adverse effect on the health of the newborn child [9, 10].

### Nutrition and infection in pregnancy

Malnutrition in pregnant, lactating women and in women of childbearing age is a common problem in sub-Saharan Africa (SSA) and is attributable to limited weight gain during pregnancy and micronutrient deficiencies [11, 12]. In Rwanda, anemia and low hemoglobin levels are present in 19% women of childbearing age, of which 7% are underweight [13, 14]. In a recent study, during early pregnancy, anemia was found to be present in 33% participants; in addition, participants showed deficiencies in iron levels as measured by ferritin and soluble transferrin receptors (sTfRs) (19.1%) [15]. In the same study, 27.3% and 7.9% participants showed high levels of inflammatory markers; C reactive protein (CRP) and alpha A glycoprotein (AGP) suggestive of infection or other inflammatory processes [15].

Systemic, sexually transmitted or genitourinary infections are common in SSA populations [16, 17]. In Rwanda, hepatitis B virus, co-infection with hepatitis B virus/human immunodeficiency virus (HIV), and syphilis affects 3.5%, 4.1%, and 2% pregnant women, respectively [18]. Other infectious diseases such as malaria affect 5.7% of pregnant women [19]. Overall malaria burden is high with 403 cases per 1,000 in 2016 and annual incidence increasing since 2012 [20]. Maternal systemic inflammation during pregnancy has been associated with diverse adverse outcomes including fetal growth restriction, and preterm birth [1, 21, 22], but little is known about its association with maternal leukocyte telomere length, particularly in SSA, where malnutrition and infection during pregnancy are more common than North American or European contexts.

To the best of our knowledge, no studies have investigated the relationship between maternal nutritional status, infection, and maternal leukocyte telomere length in SSA. In this study, we evaluated the association between nutritional and infectious disease risk factors and leukocyte telomere length during early pregnancy in women from Gasabo District, Rwanda.

## Methods

### Participants and inclusion criteria

We assessed leukocyte telomere length in healthy pregnant women aged 18–45 years as part of a prospective, cohort study of pregnant women from 10 health centers in Gasabo District, Kigali Province [15]. Participants were enrolled from September to October 2017 as previously described [15] and were in early pregnancy (gestational age 9–15 weeks, as confirmed by ultrasound and self-reported last menstrual period). Inclusion criteria included testing negative for HIV and syphilis, singleton pregnancy, speaking Kinrywanda or English and providing written consent for participation. Out of 420 participants, 300 were included in the telomere substudy based on convenience sampling. Two women were excluded due to unclear labeling of the samples and 1 did not complete the study due to loss to follow-up. Leukocyte length was assessed in 297 participants. The participating health centers were categorised into rural or urban and each contributed 50% of the participants.

### Recruitment procedure and ethical approval

Pregnant women were approached by community health workers in charge of maternal and child healthcare in the catchment area under their responsibilities as previously described in detail [15]. Eligible candidates reported to the nearest health center for preliminary screening, and trained enumerators obtained verbal and written informed consent from participants prior to their inclusion in the study.

The Institutional Review Board (IRB) of the University of Rwanda and Committee on Human Research (CHR) at the University of California, San Francisco, approved the study protocol.

### Data collection

After participant enrollment, trained phlebotomists collected whole blood samples, and six trained interviewers conducted verbal interviews to collect data on sociodemographic parameters and performed anthropometric measurements. Four on-site laboratory technicians separated blood for micronutrient analyses and processed laboratory specimens.

### Infection and nutritional assessment

Serum and whole blood samples were frozen at − 80 °C at the University Teaching Hospital of Kigali before shipping them to VitMin Lab (NutriSurvey) in Germany for analyses of micronutrients and inflammatory markers. Using the combined sandwich enzyme-linked immunosorbent assay technique [23], samples were analyzed for sTfRs, RBP, ferritin, AGP, and CRP. For data interpretation, we used cutoff point values recommended by the World Health Organization (WHO): anemia (hemoglobin < 11 g/dL) [24], low serum ferritin < 12 µg/L, and RBP < 0.83 µmol/L. Further, serum concentrations of > 5 mg/L CRP and > 1 AGP served as acute and chronic markers of inflammation, respectively [25].

#### Leukocyte telomere length analysis

Whole blood samples were shipped to the Elizabeth Blackburn Laboratory at the University of California, San Francisco, for leukocyte telomere length analysis. DNA was extracted using the QIAamp® DNA Investigator Kit (cat. no. 56,504; QIAGEN), and leukocyte telomere length was determined using quantitative PCR. The assay to measure telomere length was an adaptation of the original method published by Cawthon [26], as presented by Lin et al. [27]. Telomere length is expressed as T/S, representing the ratio of a telomeric product to a single-copy gene product [27]. The average coefficient of variation for these samples were 2.1%.

### Screening for genitourinary infections

Vaginal swabs were collected from all participants and examined for genitourinary infections. Cultures were grown and biochemical identification was performed, according to standard procedures [28]. As previously described [15], *Trichomonas vaginalis* presence was determined using wet mount microscopy [29], while *Candida albicans* presence was determined by cultures, followed by the germ tube test [30]. *Chlamydia trachomatis* was detected using CORTEZ One-Step Chlamydia Rapicard™ [31]. The culture method used for detecting *Neisseria gonorrhea* was not successful.

Urinary tract infection was diagnosed by growing cultures using standard procedures [32], and the final identification process involved using the catalase test and analytical profile index (API 20E) [33].

### Demographic, dietary, and anthropometric data

Questionnaires were administered to collect demographic data, including maternal age, education level, residence, socioeconomic status, partnership/marital status, and reproductive history. The questionnaire covered food frequency items necessary to evaluate the Minimum Dietary Diversity for Women [34, 35].

A standardized digital scale and portable stadiometer were used to measure the weight and height, respectively, of participants, and body mass index (BMI) was then calculated. Following the WHO and Centers for Disease Control guidelines [36], BMI was used to categorize women as follows: <18.5 kg/m^2^ = underweight; 18.5 to < 25 kg/m^2^ = normal weight; 25.0 to < 30 kg/m^2^ = overweight; and ≥ 30.0 kg/m^2^ = obese. The mid-upper arm circumference (MUAC) was recorded, which served as an indirect indicator of nutritional status. MUAC < 23 cm indicated nutritional deficiency, while MUAC > = 23 cm indicated nutritional sufficiency [37].

### Statistical analysis

**Fig. 1:**
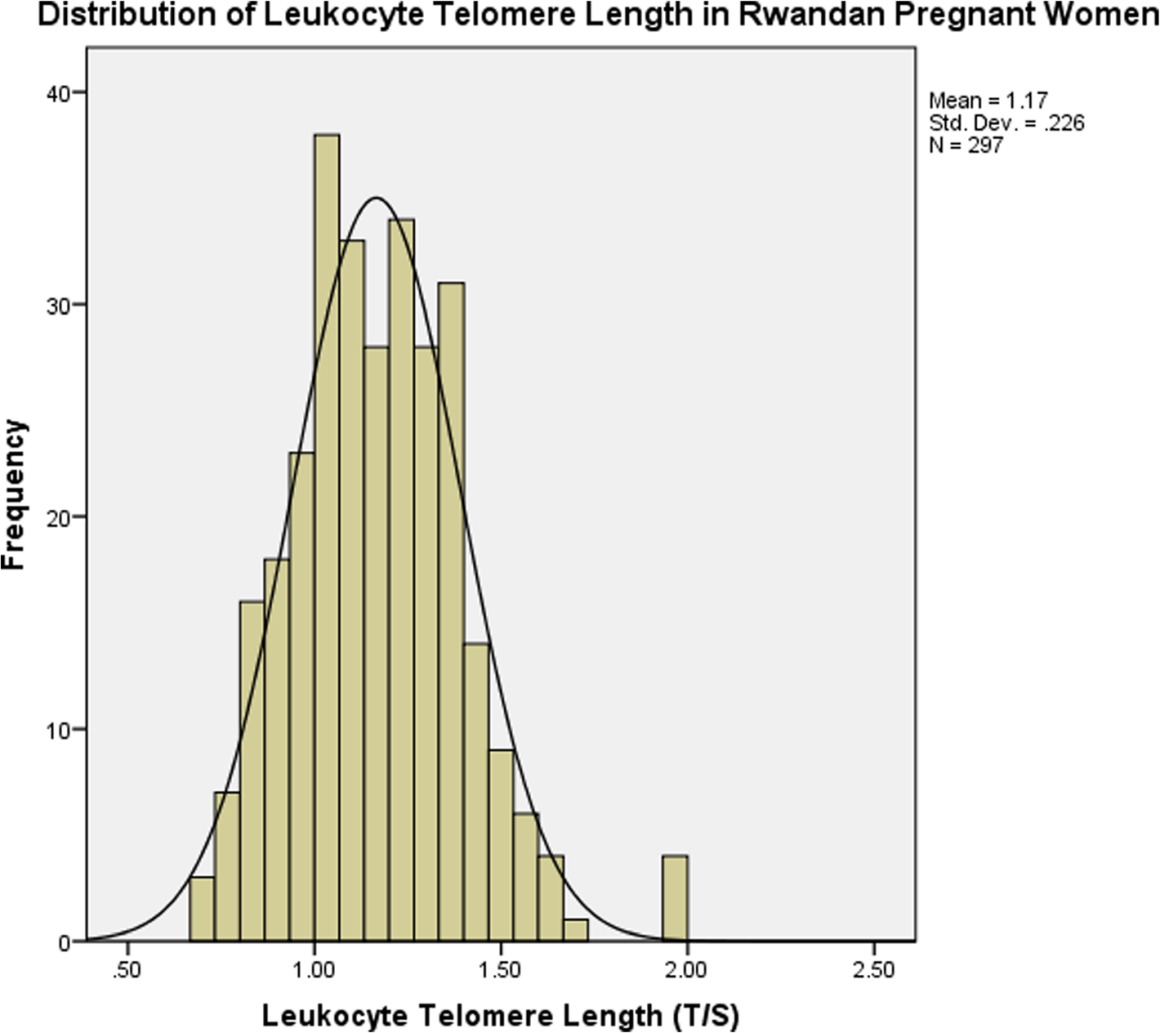
Distribution of Leukocyte Telomere Lenght among pregnant women in Rwanda

Data were checked for consistency using Excel 16.5 and then coded and exported to SPSS v20 for analyses. Normality of continuous variables was graphically assessed using Quantile-Quantile (QQ), Probability-Probability (PP) plots and histograms. Test indicated that leukocyte telomere length has a normal distribution (Fig. 1). Simple and multiple linear regressions were used to assess possible relationships for all predictors, including maternal nutrition and infectious disease status with maternal leukocyte telomere length. Means and standard deviations were calculated for predictors of interest. All variables with significance at p ≤ 0.15 were included in multivariable models. Backward stepwise regression was used, with significance set at *p* < 0.05; similarly, multivariable models had significance set at *p* < 0.05. Variables that were collinear (*r* > 0.7) were not included in the models concurrently, including presence of *Chlamydia trachomatis* bacterial infection and other sexually transmitted infections. Results are reported as slope (β) with 95% confidence interval (CI) estimated by final multivariable models using linear multivariable regression and backward stepwise technique.

## Results

### Sociodemographic, anthropometric and behavioral data and leukocyte telomere length

Mean gestational age at enrollment was 13.04 ± 3.50 weeks for 297 participants. Mean maternal age was 28.16 ± 6.10 years, and mean leukocyte telomere length was 1.16 ± 0.22 T/S. Most participants (82.2%; *n* = 244) were between 20 and 35 years, and for this age category, the mean leukocyte telomere length was 1.17 ± 0.22 T/S (Table 1). Maternal age had a significantly negative association with maternal leukocyte telomere length (β = −2.96; *p* = 0.003). No association was found between residential area within Gasabo District (urban vs. rural), occupation (employed vs. not working), and marital status (living with a partner vs. living alone) and maternal leukocyte telomere length (Table 1). Participants who had secondary schooling or higher had longer telomeres than those who did not (β = 0.17, 95% CI: 0.03–0.14; *p* = 0.003; Table 1). Primary schooling was associated with shorter leukocyte telomere length as compared with no education (Table 1).

**Table 1:** Social demographic/behavioral characteristics and leukocyte telomere length (*N* = 297)

	**Descriptive **		**Simple linear regression **		
	**Total N (%) or mean +/-SD**	**Leukoctye telomere length mean+/-SD(T/S)**	**β**	**CI**	***p***
**Age in years**	28.16 ± 6.10	1.16 ± 0.22	-2.96	[.010; -0.02]	0.003**
**Age**					
<20	13 (4.3)	1.18 ± 0.21	Ref.		
≥ 20-35	244 (82.2)	1.17 ± 0.22	-0.11	[-021; 0.06]	0.31
>35	40 (13.5)	1.11 ± 0.22	-0.14	[-013; 0.11]	0.90
**Gestational age (weeks) at recruitment **	13.04 ± 3.5		0.012	[0.01; 0.2]	0.74
**Residence **					
Urban	104 (35.0)	1.17 ± 0.21	Ref.		
Rural	193 (65.0)	1.14 ± 0.25	0.5	[-03; 0.08]	0.32
**Occupation **					
Paying Job	150 (50.5)	1.19 ± 0.23	refer		
Unemployed	147 (49.5)	1.17 ± 0.22	0.8	[-0.01; 0.05]	0.14
**Living with a partner (*n***^**a**^** = 296)**					
Yes	273 (92.2)	1.17 ± 023	Ref.		
No	23 (7.8)	1.11 ± 0.16	-0.06	[-0.15; 0.04]	0.26
**Educational level **					
Never schooled	146 (49.2)	1.15 ± 0.23	Ref.		
Primary	61 (20.5)	1.06 ± 0.18	-0.16	[-0.16; -0.3]	.005**
Secondary or higher	90 (30.3)	1.24 ± 0.21	0.17	[0.03; 0.14]	.003*
**Body Mass Index (BMI) (kg/m**^**2**^**)**	23.09 (3.39)	1.16 ± 0.22	-2.11	[-0.02; 0.01]	0.03*
**BMI (category) (*n***^**a**^** = 293)**					
Underweight	11 (3.8)	1.12 ± 0.26	Ref.		
Normal Weight	215 (73.4)	1.18 ± 0.22	0.10	[-0.06;0.17]	0.39
Overweight	55 (18.7)	1.11 ± 0.23	-0.04	[-0.15; 0.11]	0.73
Obese	12 (4.1)	1.16 ± 0.25	0.31	[-014; 0.19]	0.75
**Alcohol use (at least 2-4 times per month) **					
No	228 (76.8)	1.17 ± 0.25	Ref.		
Yes	69 (23.2)	1.16 ± 0.21	0.01	[-0.05; 0.06]	0.83
**Gender-based Violence **					
Yes	12 (4.0)	1.17 ± 0.22	Ref.		
No	285 (96.0)	1.05 ± 016	0.10	[-0.24; 0.01]	0.08

**p < 0.01, *p < 0.05

n^a^ Sample size reduced by missing responses among covariates

BMI of most participants was within the normal range (73.4%; *n* = 215), with the mean value 23.09 ± 3.39 kg/m^2^. An increase in BMI measured continuously was associated with a decrease in overall leukocyte telomere length (β = −2.11, 95% CI: −0.02 to 0.01; *p* = 0.03). No association was found between alcohol consumption and maternal leukocyte telomere length, although self-reported gender-based violence trended towards significance with shorter telomere length (β = 0.10, 95% CI: −0.24 to 012; *p* = 0.08).

### Obstetrical history, pregnancy outcomes and telomere length

The majority of the cohort was multiparous (*n* = 178, 59.9%) and had not experienced stillbirth (*n* = 289, 96.3%). Most participants had not undergone a cesarean section (*n* = 261, 90.9%), experienced previous preterm labor (*n* = 246, 82.8%), or suffered a miscarriage (*n* = 250, 85.6%). Most participants had a vaginal delivery (*n* = 239, 80.5%) with the current pregnancy.

In total, 9.1% (*n* = 27) of participants experienced preterm birth (gestational age < 37 weeks). The mean gestational age of the cohort was 38.8 ± 2.1 weeks, and for participants with preterm neonates, it was 33.6 ± 2.6 weeks. Participants who delivered before 34 weeks of gestation represented 3.7% of the cohort (*n* = 11), while those who delivered from 34 to 36 weeks of gestation represented 5.4% (*n* = 16) of the cohort. No statistically significant association was found between maternal obstetrical history or delivery-related variables, including previous preterm birth and gestational age, and maternal leukocyte telomere length (Table 2).

**Table 2:** Obstetrical history, pregnancy outcomes and leukocyte telomere length (*n*=297)

	**Descriptive**		**Simple Linear regression **		
	**Total N (%) or mean +/-SD)**	**Leukocyte telomere Length ( mean +/-SD T/S)**	**Β**	**CI**	***p***
**Maternal parity**					
Nullipara	20 (6.7)	1.23 ± 0.19	Ref		
Primipara	99 (33.3)	1.15 ± 0.21	-1.44	[-0.09; 0.01]	0.15
Multipara	178 (60.0)	1.16 ± 0.23	-1.14	[-0.16; 0.04]	0.25
**History of stillbirth**					
No	286 (96.3)	1.16 ± 0.22	Ref		
Yes	11 (3.7)	1.13 ± 0.25	-0.03	[-0.17; 0.10]	0.60
**Previous miscarriage (*n***^**a**^** = 292)**					
No	250 (85.7)	1.16 ± 0.22	Ref		
Yes	42 (14.3)	1.16 ± 0.21	-0.01	[-0.08; 0.06]	0.81
**Previous **Caesarian** Section (*n***^**a**^** = 287)**					
No previous C. Section	261 (90.9)	1.16 ± 0.22	Ref		
Previous C. Section	26 (9.1)	1.12 ± 0.19	-0.06	[-0.13; 0.04]	0.32
**Previous Preterm Labor/ birth**					
No	246 (82.8)	1.17 ± 0.22	Ref		
Yes	51 (17.2)	1.16 ± 0.23	0.01	[-0.06; 0.07]	0.84
**Mode of delivery **					
Caesarian section	58 (19.5)	1.17 ± 0.25	Ref		
Vaginal	239 (80.5)	1.16 ± 0.21	0.03	[-0.05; 0.08]	0.61
Gestational age, weeks	38.81± 2.1	1.16 ± 0.22	0.04	[-01; 0.02]	0.59
Gestational age, weeks (preterm only <37 weeks) (*n* = 27)	33.59 ± 2.61	1.15 ± 0.15	0.09	[-0.19;0.03]	0.62
**Preterm delivery (<37 weeks) **					
No	270 (90.9)	1.16 ± 0.23	Ref		
Yes	27 (9.1)	1.46 ± 0.16	-0.01	[-09; 0.08]	0.84
**Preterm delivery (<34 weeks) **					
No	286 (96.3)	1.16 ± 0.23	Ref		
Yes	11 (3.7)	1.17 ± 0.14	0.01	[-0.13; 0.14]	0.98
**Late preterm delivery (34-<37 weeks)**					
No	281 (94.6)	1.15 ± 0.17	Ref		
Yes	16 (5.4)	1.16 ± 0.23	-0.02	[-0.13; 0.09]	0.79
Birthweight (*n* = 297) (grams)	3153.22 ± 544.45	1.16 ± 0.22	-0.08	[-0.01;0.02]	0.15
Birthweight preterm (<37 weeks) (*n* = 27) (grams)	2063.33 ± 616.85	1.15 ± 0.16	0.14	[-0.06; 0.14]	0.47
Low birth weight (<2500 g)	1874.80 ± 451.84	1.18 ± 0.16	0.27	[-0.05;0.025]	0.20

n^a^ Sample size reduced by missing responses among covariates

### Inflammation and infections in pregnancy and leukocyte telomere length

Overall, 26% of the cohort (*n* = 77) had acute inflammation as suggested by elevated CRP levels and 7.4% had chronic inflammation as indicated by elevated AGP level (Table 3). There was no association between maternal leukocyte telomere length and CRP levels, AGP levels, or presence of urinary tract infections (Table 3). Having a sexually transmitted infection during pregnancy and shorter maternal telomere length approached statistical significance (β = −0.104, 95% CI: −0.102 to 0.005; *p* = 0.07), as did having a bacterial *Chlamydia trachomatis* infection (β = −0.010, 95% CI: −0.12 to 0.01; *p* = 0.08; Table 3).

**Table 3:** Maternal inflammation, infection and leukocyte telomere length (*n*=297)

	**Descriptive **		**Simple Linear regression **		
	**Total N (%) or mean ±SD)**	**Leukocyte telomere Length mean ±SD (T/S)**	**Β**	**CI**	***P***
**Markers of Acute and Chronic Inflammation**					
**C Reactive Protein (CRP mg/l) **	5.08 ± 12.33	1.16 ± 0.22	0.02	[-0.01; 0.03]	0.67
**CRP(>5 mg/l) (*n***^**a**^** = 296)**					
No	219 (74.0)	1.16 ± 0.23	ref		
Yes	77 (26)	1.17 ± 0.21	0.04	[-0.04; 0.07]	0.53
**α**_**1**_**-acid glycoprotein (AGPg/dl)**	0.61 ± 0.41	1.16 ±0.22	-0.45	[-0.07; 0.04]	0.64
**α**_**1**_**-acid glycoprotein (AGP>1 g/l) **					
No	275 (92.6)	1.17. ±0.23	ref		
Yes	22 (7.4)	1.13 ± 0.15	-0.04	[-0.13; 0.06]	0.48
**Presence of Infectious Disease**					
**Urinary Tract Infections (UTI) **					
No	259 (87.2)	1.17 ± 0.23	Ref		
Yes	38 (12.8)	1.16 ± 0.16	-0.01	[-0.08; 0.07]	0.84
**UTI strain (*n* = 38)**					
*Stenotrophomonas maltophilia*	2 (5.3)				
*Escherchia coli*	22 (57.9)				
*Klebsialla pneumoniae*	11 (28.9)				
*Staphylococcus epidermitis*	3 (7.9)				
**Sexually Transmitted Infection (STI)**					
**No **	189 (63.6)	1.18 ± 0.23	ref		
**Yes **	108 (36.4)	1.13 ± 0.22	-0.104	[-0.102; 0.005]	0.07
**Type of STI **					
**Chlamydia trachomatis**					
No	235 (79.1)	1.17 ± 0.22	ref		
Yes	62 (20.9)	1.12 ± 0.25	-010	[-0.12; 0.01]	0.08
**Trichomonas vaginalis**					
No	282 (94.9)	1.16 ± 023	ref		
Yes	15 (5.1)	1.17 ± 0.19	0.002	[-0.11; 0.12]	0.99
**Candida albicans **					
No	233 (78.5)	1.17 ± 0.23	ref		
Yes	64 (21.5)	1.14 ± 0.19	-0.51	[-0.09; 0.03]	0.25

n^a^ Sample size reduced by missing responses among covariates

### Nutrition in pregnancy and leukocyte telomere length

A significant number (*n* = 92, 31%) of participants had anemia during pregnancy; 3.4% (*n* = 10) with a lower percentage having iron deficiency as indicated by ferritin or soluble transferrin receptors deficiency (Table 4). Nineteen-point two percent of participants had ferritin deficiency (*n* = 58) with 3.4% (*n* = 10) having transferrin receptor deficiency (Table 4). Continuous measures of sTfR levels were inversely associated with telomere length (β = −0.13, 95% CI: −0.03 to 0.01; *p* = 0.02), as were RBP levels (β = −2.19, 95% CI: −0.13 to 0.01; *p* = 0.03). Deficiencies in sTfR levels (> 8.3 mg/L) approached statistical significance, with a negative association between deficiency and longer leukocyte telomere length (β = −0.10, 95% CI: −0.26 to 0.17; *p* = 0.08; Table 4). No association was present for other markers of maternal nutritional health, including RBP deficiency, ferritin deficiency, inadequate dietary diversity, indicators of malnutrition and maternal leukocyte telomere length (Table 4).

**Table 4:** Maternal nutrition and leukocyte telomere length (*n*=297)

	**Descriptive**		**Simple Linear regression**		
	**Total N (%) or mean ±SD)**	**Leukocyte telomere length mean ±SD (T/S)**	**β**	**CI**	***P***
**Hemoglobin (g/dl)**	11.23 ± 1.13	1.16 ±0.22	0.04	[-0.01; 0.03]	0.47
**Anemia (<11 g/dl) **					
No	205 (69.0)	1.15 ± 0.21	Ref		
Yes	92 (31.0)	1.19 ± 0.24	0.07	[-02;0.09]	0.21
_***S***_**TFR (mg/L) (Soluble Transferrin Receptors) **	4.81 ± 1.49	1.16 ±0.22	-0.13	[-0.03; -0.01]	0.02*
_***S***_**TFR (mg/L) Deficiency**					
No deficiency **(≤8.3 mg/L)**	287 (96.6)	1.17 ± 0.22	Ref		
Deficiency **(>8.3 mg/L)**	10 (3.4)	1.04 ± 0.18	-0.10	[-0.26; 0.17]	0.08
**Retinol Binding Protein (RBP) (μmol/L)**	1.38 ± 0.40	1.16 ± 0.22	-2.19	[-0.13; -0.01]	0.03*
**RBP(μmol/L) Deficiency**					
No deficiency **(**≥0.83 μmol/L**)**	239 (80.5)	1.17 ± 0.18	Ref		
Deficiency **(**< 0.83 μmol/L**)**	58 (19.5)	1.16 ± 0.23	0.03	[-0.05; 0.08]	0.66
**Ferritin (μg/L)**	73.52	1.16 ± 0.22	-0.09	[-0.01; 0.03]	0.12
**Ferritin Deficiency (<12** μg/L**) **					
No Deficiency (<12 μg/L)	240 (80.8)	1.16 ± 0.22	Ref		
Deficiency** (≥12** μg/L**)**	58 (19.2)	1.17 ± 0.23	-0.01	[-0.07; 0.06]	0.84
**Minimum Dietary Diversity for Women (MDDW)**	4.61 ± 1.68	1.16 ± 0.22	-0.03	[-0.06; 0.3]	0.56
**MDDW Deficiency**					
Adequate MDDW	145 (48.8)	1.15 ± 0.22	Ref		
Low MDDW	152 (51.2)	1.17 ± 0.23	-0.03	[-0.07; 0.04]	0.50
**Mid-Upper Arm Circumference (MUAC) d(cm)(Mean +/-SD)**	25.72 ± 3.06	1.16 ± 0.23	-0.09	[-0.02; 0.002]	0.30
**MUAC Indication of Malnutrition**					
≥23 cm	236 (79.5)	1.16 ± 0.23	Ref		
<23 cm	58 (19.5)	1.18 ± 0.22	-0.04	[-0.09; 0.04]	0.45

*p < 0.05

n^a^ Sample size reduced by missing responses among covariates

### Multivariable linear regression

Independent predictors of longer leukocyte telomere length included younger maternal age (β = −0.14, 95% CI: −0.01 to − 0.001; *p* = 0.01), no schooling vs. primary schooling (β = −0.19, 95% CI: −0.17 to − 0.041; *p* = 0.002), lower levels of sTfRs (β = −0.15, 95% CI: −0.04 to − 0.007; *p* < 0.01), and lower levels of ferritin (β = −0.13, 95% CI: −0.01 to − 0.001; *p* = 0.02; Table 5). Similarly, in the backward stepwise regression model, independent predictors of longer telomere length were younger age (β = −0.15, 95% CI: −0.01 to − 0.001; *p* < 0.01), absence of primary schooling (β = −0.21; 95% CI: −0.18 to − 0.05; *p* < 0.01), lower levels of sTfRs (β = −0.15, 95% CI: −0.04 to − 0.007; *p* < 0.01), lower levels of ferritin (β = −0.12, 95% CI: −0.01 to − 0.001; *p* = 0.027), and lower levels of RBP (β = −0.11, 95% CI: −0.12 to − 0.001; *p* = 0.04; Table 5).

**Table 5:** Multiple linear regression for determinants of maternal leukocyte telomere length (*n*=297)

	**Linear regression model full model **				**Adjusted model **			
	**Β**	**SE**	**CI**	***P***	**β**	**SE**	**CI**	***P***
**Age (years)**	-0.140	0.02	[-0.01; -0.001]	0.01	-0.15	0.02	[-0.01; -0.001]	0.008**
**Occupation **								
Paying Job				ref				
Housewife	-0.002	0.025	[-0.05; 0.048]	0.97	-	-	-	-
**Education Level **								
Never schooled				ref				
Primary	-0.190	0.033	[-0.17; 0.041]	0.002	-0.21	0.033	[-0.18; -0.05]	0.001**
Secondary or higher	0.122	0.030	[-0.001; 0.12]	0.045	0.11	0.109	[-0.004; 0.11]	.070
**Body Mass Index (BMI) kg/m**^**2**^	-0.094	0.004	[-0.014; 0.01]	0.101	-	-	-	-
**Gender Based Violence (GBV)**								
Yes				ref				
No	-0.107	0.063	[-0.25; 0.01]	0.053	-	-	-	-
**Parity Nullipara(reference)**				ref	-	-	-	-
Primipara	-0.020	0.055	[-0.125; 0.09]	0.75	-	-	-	-
Multipara	0.046	0.027	[.0.03; 0.074]	0.430	-	-	-	-
Birth weight	0.053	.0001	[-0.01; 0.001]	0.350				
**Sexually transmitted Infections **								
**No **				ref				
**Yes**	-0.071	0.026	[-0.84; 0.018]	0.202				
**sTFR (mg/L)**	-0.156	0.009	[-0.04; 0.007]	0.006	-015	0.008	[-0.04; -0.007]	0.005**
**Retinol Binding Protein (RBP) (μmol/L)**	-0.093	0.031	[-0.11; 0.010]	0.098	-0.11	0.03	[-0.12; -0.001]	0.04
**Ferritin (**μg/L)	-0.130	.0001	[-0.01; 0.001]	0.023	-0.124	0.001	[-0.01; -0.001]	0.027*

**p < 0.01, *p < 0.05

## Discussion

This is the first study to assess association between maternal infections, nutritional health and leukocyte telomere length in pregnant women in SSA. Furthermore, it is the first study of leukocyte telomere length among women in Rwanda. We found evidence of leukocyte telomere length as a marker of biological aging in Rwandan women and also of possible associations between maternal micronutrient status and leukocyte telomere length in pregnant women in Rwanda. Other leukocyte telomere length studies with pregnant women have been conducted primarily in the European and North American context, which presents a different environmental milieu in terms of overall burden of infectious disease and maternal nutritional health [38, 39]. In contrast with these other studies, the endemicity of infectious diseases in Kigali, Rwanda, including clinical and subclinical infections such as a higher prevalence of sexually transmitted infections and endemicity of malaria, provides a different context to assess the association between maternal leukocyte telomere length and birth outcomes [40].

### Maternal age, leukocyte telomere length and Rwandan women

A strong inverse association was found between maternal age and leukocyte telomere length, which is consistent with results of previous studies [4, 41, 42] demonstrating that leukocyte telomere length is a biological marker for aging in Rwandan women [41, 43]. This is the first study to our knowledge conducted of leukocyte telomere length in Rwandan women. The age range in our study was relatively narrow with the majority between 20 and 35 years of age (mean 28.16 ± 6.10) and even within this range, maternal age was highly predictive of leukocyte telomere length adjusting for other factors. Previous studies have found that Africans have longer leukocyte telomere length than Caucasians [44]; our results for reproductive age women are similar to those reported from a large population-based survey in United States for a similar age range of women and comparable to the African-Americans surveyed [45].

### Maternal nutritional status

Several micronutrient markers were independently associated with shorter maternal leukocyte telomere length, after adjusting for other predictors. Specifically, we found associations between body iron stores as measured by lower numbers of soluble transferrin receptors, ferritin levels, and longer leukocyte telomere length. However, we found no associations between anemia and iron deficiency and leukocyte telomere length. Previous findings have been mixed including a study involving American adults (> 65 years) in whom high ferritin levels were associated with shorter telomere length [46] and a large study with British adults where transferrin saturation was associated with shorter leukocyte telomere length [47]. In contrast, another study found that iron consumption, as measured by dietary intake, was associated with longer telomere length in adults [48]. Importantly, the context where our study was conducted has different environmental stressors than in the previously described studies. As malaria infection is common in East Africa, including Rwanda, lower iron levels have been found to be associated with a reduced susceptibility to malaria infection in pregnant women [49]. Lower iron levels may have indirect health benefits in the context of areas with endemic malaria or other high incidence areas.

Alternatively, increased concentrations of iron can cause oxidative stress, consequently leading to telomere shortening [50]. Iron is a redox-active transitional metal with pro-oxidant properties [51]. Excessive iron during pregnancy can lead to ROS overproduction and oxidative stress [46]. Administering iron supplements to pregnant women is common as part of antenatal care services [14]; some pregnant Rwandan women may receive iron supplements to tackle iron deficiency, eventually leading to excessive iron in the body.

We also found an inverse relationship between RBP, an indirect indicator of retinol (vitamin A) levels, and leukocyte telomere length. As vitamin A is one of the antioxidants that provides a buffer against oxidative stress, it may protect against accelerated leukocyte telomere shortening [52]. Our results were unexpected and differ from those of other studies in which retinol levels were found to be positively associated with leukocyte telomere length [48]; moreover, vitamin A supplementation has been reported to be associated with longer telomere length [53]. However, these previous studies were not conducted with pregnant women in the first trimester of pregnancy. High levels of vitamin A, particularly during the first trimester, are associated with congenital malformations of the central nervous and cardiovascular systems [54], and high levels of serum retinol during pregnancy are associated with postpartum depression [55]. Elevated serum retinol levels may thus serve as an adverse risk marker of maternal and neonatal outcomes; further studies with multiple timepoints in pregnancy are warranted to assess direct effects.

Neither maternal BMI, MUAC or dietary intake measured by the MDDW index were associated with leukocyte telomere length. Our findings differ from the results of other studies that have consistently found maternal BMI levels associated with leukocyte telomere length [56, 57] as well as those that suggest that improved nutrition is associated with longer leukocyte telomere length [47]. In this study, a low percentage of women were obese (4.1%) and underweight (3.8%), with less heterogeneity in BMI compared with other population groups potentially explaining the contradictory results. Excess adipose tissue in obesity has been associated with shorter leucocyte telomere length but in North American contexts where obesity is more prevalent [58]. Additionally, our measurements were taken during early pregnancy which could have biased findings in contrast with other studies that have use pre-pregnancy BMI. Few studies measured weight during pregnancy in resource-poor settings in relation to maternal leucocyte telomere length.

### Infections and inflammation

We found no association between biomarkers for inflammation and infection including sexually transmitted infections and leukocyte telomere length, although in bivariate analyses, any presence of sexually transmitted infection and infection with chlamydia trachomatis approached statistical significance for shorter leukocyte telomere length. Previous studies of infectious disease, specifically HIV in South Africa, have found shorter leukocyte telomere length with infection [59] although other studies with HIV infected individuals did not find any association, primary in the context of antiretroviral therapy immune reconstitution [38, 60]. Additional studies evaluating multiple pathogens have found that the association between shorter leukocyte telomere and infection may be pathogen specific [61]. We also assessed the association between general markers of inflammation and leukocyte telomere length as these have previously been found associated with shorter telomere length [39]. However, we did not assess duration or timing of infection and as such we may have not had sufficient information to assess associations with leukocyte telomere length. CRP levels rise sharply during the early phase of an infection, whereas AGP levels gradually increase so timing of testing may be critical to assess levels [62]. Furthermore, other studies were not conducted with pregnant women and CRP levels increase and AGP decrease with duration of gestation [21]. As such, the interplay between inflammatory status and leukocyte telomere length may not be fully understood in the context of pregnancy. Alternatively, it is possible that the cutoff points used for active and chronic infections may not be appropriate for developing countries with a high infectious disease burden [63], such as Kigali, Rwanda. Elevated CRP levels were lower than those reported in pregnant Kenyan population and AGP slightly higher, although the gestational age range for the group including women in later stages of pregnancy potentially accounting for the differences [64]. Another study with Ugandan women at similar timing of gestation had comparable elevated AGP levels but lower CRP ones [65].

### Maternal sociodemographics

Maternal education level (primary schooling vs. no schooling) was inversely associated with maternal leukocyte telomere length. Previous studies involving pregnant women have reported that higher education level is associated with longer leukocyte telomere length and is a potential good proxy for socioeconomic status [66]. It remains unclear as to why primary education was associated with shorter leukocyte telomere length in our study population after adjusting for age and other risk factors. It is possible that unknown confounders are associated with higher maternal education levels that additionally adversely impact leukocyte telomere length in Rwandan women. Further studies are warranted to comprehend the relationship between education level and health outcomes in East African populations.

### Maternal obstetrical history

We found no association among birth outcomes, including birth weight, low birth weight, and preterm birth, maternal reproductive history, and maternal leukocyte telomere length. Few studies have investigated these associations, but to our knowledge, none have been conducted in SSA. A study involving European mothers reported an association between shorter telomere length and intrauterine growth restriction [67], and previous studies have reported mixed results on the association between gravidity, parity, and maternal telomere length [68, 69].

## Conclusions

We found associations between maternal age and micronutrients levels in early pregnancy and leukocyte telomere length in Rwandan women. Specifically, similar to other studies, we found that older maternal age was associated with shorter leukocyte telomere length even within a relatively narrow age range for women of reproductive age. We additionally found an association between markers of serum iron levels including higher ferritin levels and transferrin saturation receptor counts and shorter telomere length as well as lower retinol binding protein levels and shorter telomere length possibly suggestive of maternal health in pregnancy or alternatively specific to the environmental context of Rwanda. As the relationship between nutrition and infectious disease involves a feedback loop with infections affecting nutritional status and nutritional deficiencies impacting immune response and susceptibility to infections; further studies in this area are needed in developing countries.

## Limitations

Ours is the first study to assess leukocyte telomere length in Rwanda. Additional studies are needed with more diverse population groups including men and children. Studies with pregnant women should more comprehensively explore maternal nutritional health and leukocyte telomere length in terms of nutritional assessments, including analyzing the intake of vitamin D, selenium, vitamin C, and other vitamins and minerals, in relation to maternal leukocyte telomere length outcomes. Future studies should also include multiple measurements of leukocyte telomere length during pregnancy, in addition to neonatal assessments and multiple nutritional and anthropometric maternal measurements throughout different periods of gestation.

Studies should also investigate the impact of pre-pregnancy BMI and maternal weight gain during pregnancy on maternal and neonatal outcomes including leukocyte telomere length as an outcome. Other studies should also comprehensively investigate infectious disease history including history of infection with malaria, STI history and other infectious disease burden including tuberculosis, hepatitis and dengue.

## Public health implications

Nutritional intake during pregnancy is an actionable area for public health interventions in Rwandan women and children. Thus, further studies should assess the relationship between high iron levels, retinol binding protein and other markers of vitamin A status and shorter maternal leukocyte telomere length in the context of endemic infectious diseases. It is possible that the current regimens of iron and multivitamin supplementation during pregnancy do not confer the same benefits in all pregnant women, particularly those living in areas with a high incidence of malaria or a high burden of other infectious diseases.

## Data Availability

Available with the corresponding author (etiennen70@gmail.com) and will be deposited in a public repository as soon as we gain permission to do so.

## Abbreviations

AGP: α1-acid glycoprotein
BMI: Body mass index
CI: Confidence interval
CRP: C-reactive protein
HIV: Human immunodeficiency virus
MUAC: Mid-upper arm circumference
RBP: Retinol-binding protein
ROS: Reactive oxygen species
SSA: Sub-Saharan Africa
sTfR: Soluble transferrin receptor
WHO: World Health Organization
